# Recreational drug use and use of drugs associated with chemsex among HIV-negative and HIV-positive heterosexual men and women attending sexual health and HIV clinics in England

**DOI:** 10.1016/j.drugpo.2020.103101

**Published:** 2021-05

**Authors:** Ada R Miltz, Alison J Rodger, Janey Sewell, Richard Gilson, Sris Allan, Christopher Scott, Tariq Sadiq, Paymaneh Farazmand, Jeffrey McDonnell, Andrew Speakman, Lorraine Sherr, Andrew N Phillips, Anne M Johnson, Simon Collins, Fiona C Lampe, AURAH and ASTRA Study Groups

**Affiliations:** a Institute for Global Health, University College London, London, UK; b City of Coventry Healthcare Centre, Coventry, UK; c West London Centre for Sexual Health, London, UK; d Courtyard Clinic, St George's Healthcare NHS Trust, London, UK; e Princess Royal Community Health Centre, Huddersfield, UK; f Department of Clinical, Educational and Health Psychology, University College London, London, UK; g HIV i-Base, London, UK

**Keywords:** Recreational drug use, Chemsex, Depression, Anxiety, Sexual behavior, Heterosexual

## Abstract

**Background:**

There is little information on the prevalence of recreational drug use among UK heterosexual men and women, in particular on use of drugs associated with ‘chemsex’ within gay communities. The aim of this study was to examine among HIV-negative and HIV-positive heterosexual men and women in England: (i) the prevalence of recreational drug use (including use of drugs associated with chemsex), (ii) socio-economic/lifestyle correlates of drug use, and (iii) the association of drug use with sexual behavior measures and mental health symptoms.

**Methods:**

Data are from the AURAH study of HIV-negative individuals attending sexual health clinics across England (2013–2014) and the ASTRA study of HIV-positive individuals attending HIV outpatient clinics in England (2011–2012). Prevalence of recreational drug use (past three months) and associations are presented separately among the four sample groups: HIV-negative (*N* = 470) and HIV-positive (*N* = 373) heterosexual men and HIV-negative (*N* = 676) and HIV-positive (*N* = 637) women.

**Results:**

The age standardized prevalence of any drug use was 22.9%, 17.1%, 15.3%, and 7.1% in the four sample groups respectively. In all groups, cannabis was the drug most commonly used (range from 4.7% to 17.9%) followed by cocaine (1.6% to 8.5%). The prevalence of use of drugs associated with chemsex was very low among HIV-negative participants (1.0% heterosexual men, 0.2% women) and zero among HIV-positive men and women. In age-adjusted analysis, factors linked to drug use overall and/or to cannabis and cocaine use specifically in the four sample groups included Black/mixed Caribbean and white (vs. Black/mixed African) ethnicity, lower level of education , cigarette smoking, and higher risk alcohol consumption. Associations of recreational drug use with measures of condomless sex, depression, and anxiety were observed in the four groups, but were particularly strong/apparent among women.

**Conclusion:**

Providers need to be aware of cannabis and cocaine use and its potential link with sexual risk behavior and symptoms of depression and anxiety among heterosexual men and women attending sexual health and HIV clinics.

## Introduction

In the Crime Survey for England and Wales (2017/2018), a population-based study, the prevalence of any recreational drug use in the past year was 11.8% for men and 6.2% for women overall, and 20% among men and women aged 16 to 24 years (ONSFlatley, 2018). The use of recreational drugs has been linked to depressive symptoms, sexual risk behavior and increased risk of HIV infection (Scott et al., 2007). In a recent meta-analysis of seven studies (one in the UK), cannabis use during adolescence was associated with depression in young adulthood (pooled OR 1.37 [95% CI: 1.16, 1.62]) (Gobbi et al., 2019). In the British National Survey of Sexual Attitudes and Lifestyles (Natsal, 2010–2012), past year use of illicit drugs was strongly associated with reporting two or more condomless sex (CLS) partners in the past year among both men (OR 5.50 [95% CI: 3.61, 8.39]) and women (OR 5.24 [95% CI: 3.07, 8.94]), adjusting for age (Paquette et al., 2017), but information on type of drug used was not available. There is a need for drug-specific prevalence estimates and information on associations with sexual behavior and mental health measures among heterosexual men and women in the UK. This includes people living with HIV, for whom there are very limited data.

There is also a need to investigate whether the use of drugs associated with chemsex, which have been trending within gay communities over the past decade, have also gained popularity among heterosexual men and women in the UK. The phenomenon of chemsex has been documented since 2013 (Stuart, 2019). It involves the use of one or more psychoactive substances (mephedrone, gamma-hydroxybutyrate/gamma-butyrolactone [GHB/GBL] and/or methamphetamine) during or immediately before sex (Bourne, Reid, Hickson, Torres Rueda, & Weatherburn, 2014). These drugs tend to impact on sexual arousal, euphoria, and disinhibition to a greater extent than other commonly used recreational drugs, with implications for increased risk of STI/HIV transmission. Qualitative work suggests that chemsex is practiced in order to facilitate different/new sexual practices and prolong sexual episodes (Bourne, Reid, Hickson, Torres Rueda, & Weatherburn, 2014).

A growing number of survey studies have examined the prevalence of chemsex or use of drugs associated with chemsex among HIV-negative and HIV-positive gay, bisexual and other men who have sex with men (GBMSM) in Europe (Daskalopoulou et al., 2014, 2017; Druckler, van Rooijen & de Vries, 2018; Glynn et al., 2018; Hegazi et al., 2017; Ottaway, Finnerty, Buckingham & Richardson, 2017; Sewell et al., 2018, 2017; Tomkins et al., 2018). Use of drugs associated with chemsex was reported by over a fifth of 1484 HIV-negative GBMSM attending sexual health clinics in the AURAH study in England (2013–2014), and was very strongly associated with group sex and CLS (Sewell et al., 2017). Similar findings have been observed in other recent studies in Europe. There is also some evidence that the prevalence of drug use associated with chemsex is increasing among GBMSM (Hampel et al., 2019; Sewell et al., 2018).

The extent to which the types of drugs associated with chemsex within gay communities are used among heterosexual men and women is uncertain. It remains unclear whether harm reduction messaging surrounding use of drugs associated with chemsex should also target heterosexuals. This is a particularly pertinent issue given the recent rise in gonorrhoea, syphilis and chlamydia diagnoses among heterosexual men and women in England (Mitchell et al., 2020). It is acknowledged however, that the phenomenon of chemsex emerged as a means by which to facilitate enjoyment of gay sexuality against the backdrop of a heteronormative and homophobic social environment (Stuart, 2019). In many respects the practice of chemsex may be unique to gay communities. Among GBMSM, investigating the use of drugs associated with chemsex is thought to be a good reflection of the practice of chemsex itself (Stuart, 2013). However, this may not be the case among heterosexual populations, for whom these drugs may be used outside of a sexual setting.

This analysis uses data from two cross-sectional studies in England and investigates among HIV-negative and HIV-positive heterosexual men and HIV-negative and HIV-positive women separately: (i) the prevalence of recreational drug use (any, individual drugs, multiple drugs, and use of drugs associated with chemsex within gay communities), (ii) the association of socio-economic and lifestyle factors with recreational drug use (any and the two most common drugs), (iii) the relationship of recreational drug use (any and the two most common drugs) with symptoms ofdepression and anxiety, and (iv) the relationship of recreational drug use (any and the two most common drugs) with sexual behavior/attitudes.

## Methods

In this paper, data were analysed from two separate cross-sectional questionnaire studies in England. The Attitudes to and Understanding of Risk of Acquisition of HIV (AURAH) study recruited HIV-negative or unknown HIV status men and women (hereafter referred to as HIV-negative) from 20 sexual health clinics in England between June 2013 and November 2014. Individuals aged 18 years or older without diagnosed HIV were eligible for inclusion. Methodological details have been published elsewhere (Sewell et al., 2016). The Antiretrovirals Sexual Transmission Risk and Attitudes (ASTRA) study recruited HIV-positive men and women aged 18 years or older from eight HIV outpatient clinics in England between February 2011 and December 2012. Methodological details have been published elsewhere (Speakman et al., 2013). All measures used in this analysis were obtained from the study questionnaire, which was self-administered in both studies.

### Population groups under investigation

In AURAH, men who reported being heterosexual/straight and did not report anal sex with a man in the past three months, nor disclose to their family, friends or workmates as being gay, bisexual and/or attracted to men, were included in the heterosexual men category (*N* = 470). All women were analysed together (*N* = 676) regardless of their sexual orientation/sexual behavior due primarily to the very small number of gay/lesbian women in this study (*N* = 3). In ASTRA, men who reported being heterosexual/straight and did not report anal sex with a man in the past three months were included in the heterosexual men category (*N* = 373). The question on disclosure of sexual orientation to family, friends and workmates was not asked in the ASTRA questionnaire. Again, all women were analysed together (*N* = 637).

### Recreational drug use

The questions on drug use were identical in AURAH and ASTRA. Participants were asked to report whether they had used recreational drugs in the past three months and, if so, to select which drug or drugs from the following list of 18 options: acid/LSD/magic mushrooms; anabolic steroids; cannabis (marijuana, grass); cocaine (coke); crack; codeine; crystal meth (methamphetamine); Ecstasy (E, MDMA); GHB (liquid ecstasy, GBL); heroin, ketamine (K); khat (chat); mephedrone; morphine; opium; poppers (amyl nitrate, nitrites); speed (amphetamine); viagra (erectile dysfunction drugs) (and other). Other drugs specified were coded to the above categories where appropriate. Participants were also asked whether in the past three months they had injected recreational drugs (e.g. heroin, crystal meth).

Three composite measures of recreational drug use were defined:

(i) Any recreational drug use: use of at least one of the 18 drugs listed above in the past three months.
(ii) Poly drug use: use of at least three different recreational drugs (from the above list of 18) in the past three months. Information was not collected on whether these drugs were taken concurrently.
(iii) Use of drugs associated with chemsex within gay communities: use of one or more of mephedrone, methamphetamine or GHB/GBL in the past three months. It should be noted that the questionnaires did not ask about drug use during sex specifically.

### Sexual behavior and attitudes

From the questions in AURAH and ASTRA, four comparable measures of sexual behavior and attitudes were derived:

(i) CLS with a non-regular partner in the past three months, defined as report of more than one CLS partner or one CLS partner who was not a long-term partner.
(ii) CLS with two or more partners in the past three months.
(iii) Self-efficacy for sexual safety: ‘I feel confident that, if I want to, I can make sure a condom is used during sex with any partner, in any situation.’ If a participant did not strongly agree with this statement (i.e. responded with tend to agree, tend to disagree, strongly disagree, undecided/no opinion/not relevant to me, or had a missing response) this was considered to indicate low self-efficacy for sexual safety. This categorisation was chosen due to the high proportion of participants who agreed with this statement, therefore, it was believed appropriate to separate participants who specified ‘tend to agree’ from those who specified ‘strongly agree’.
(iv) Transactional sex for money or drugs in the past three months. Information on the gender of the client was not obtained.

### Symptoms of depression and anxiety

In both studies, the Patient Health Questionnaire (PHQ-9) and Generalized Anxiety Disorder Assessment (GAD-7) were used to collect information on depressive symptoms and anxiety symptoms respectively (Kroenke, Spitzer & Williams, 2001; Kroenke, Spitzer, Williams, Monahan & Lowe, 2007). A total score of 10 or greater on PHQ-9 was considered to indicate clinically significant depressive symptoms, according to the standard definition. Similarly, the standard definition of a total score of ≥10 on the GAD-7 was used to define clinically significant anxiety symptoms.

### Statistical analysis

The prevalence of individual drug use and composite measures were presented for (a) HIV-negative participants in the AURAH study and (b) HIV-positive participants in the ASTRA study, separately for heterosexual men, women and GBMSM (the latter has already been reported (Daskalopoulou et al., 2014) and is shown here for comparison). In order to facilitate comparison of drug use prevalence in AURAH and ASTRA, estimates are presented standardized for age. The age-standardized prevalence is the weighted average of drug use prevalence in each age group (<35, 35–44, 45+), using the age distribution in AURAH and ASTRA combined to obtain the weights (38.6% <35; 27.8% 35–44; 33.6% 45+).

The remainder of the paper focuses on heterosexual individuals only, with all analyses conducted in the four sample groups separately: HIV-negative and HIV-positive heterosexual men, and HIV-negative and HIV-positive women.

Associations of socio-economic and lifestyle factors with any drug use and the two most common drugs taken were assessed among the four sample groups. Given the low prevalence of poly drug use and use of drugs associated with chemsex in our samples, these measures were not investigated further. For univariable analysis, *χ*^2^ tests, *χ*^2^ tests for trend and (when expected numbers were small) Fisher's exact tests were used to compare drug use between subgroups. Modified Poisson regression with a robust variance estimator was used to produce prevalence ratios (PRs) adjusted for age (as a continuous variable) (Zou, 2004).

Associations of any drug use and the two most common drugs taken with symptoms of depression and anxiety, and with measures of CLS and self-efficacy for sexual safety were assessed. Using modified Poisson regression the following key socio-demographic factors were adjusted for: age (continuous variable), ethnicity (Black/mixed African or other ethnicity), university education (yes or no/missing), ongoing relationship (yes or no/missing), and London clinic site (London or outside London). For some outcome measures, prevalence was low and only unadjusted associations are presented. For HIV-positive sample groups this was the case for all measures of CLS, therefore findings related to this group are presented in the text only.

The proportion of missing values was <5% for all variables used in analyses. Missing values were incorporated into specific categories for the measures of drug use, depression and anxiety symptoms, CLS, university level of education, being employed, ongoing relationship, current smoking and higher risk drinking (missing was taken to mean the absence of the factor). For all other variables, participants with missing data were excluded from analysis. A sensitivity analysis was undertaken excluding missing values when defining all variables. The findings were very similar to the main analysis (data not shown). Analyses were performed by STATA statistical software (StataCorp, 2009) and were reported according to the STROBE guidelines (STROBE, 2009).

## Results

In total, 2630 individuals participated in the AURAH study (response rate was 60% of eligible patients approached), of whom 470 were heterosexual men and 676 were women. On the self-reported questionnaire, all participants reported that they had not been diagnosed with HIV. Overall, 346 heterosexual men and 446 women had an HIV test on the day of the AURAH questionnaire. Four heterosexual men and one woman tested HIV-positive. These five individuals were retained in the sample for analyses, as they were not diagnosed with HIV at the time of questionnaire completion. For heterosexual men and women respectively, 48.4% and 48.7% were of Black or mixed African ethnicity, 11.6% and 17.1% of Black or mixed Caribbean ethnicity, 31.4% and 24.4% of white ethnicity, and 8.6% and 9.8% of any other ethnicity. The median age was 29 (interquartile range IQR 25–37) and 26 (IQR 22–32) years for heterosexual men and women respectively. Overall, 44.0% of heterosexual men and 44.2% of women were born in the UK, 58.3% and 56.3% respectively had a university degree, and 74.5% and 60.8% were attending a clinic in London (Tables 1 and 2).

**Table 1:** Unadjusted and adjusted associations of demographic, socio-economic and lifestyle factors with three measures of recreational drug use in the past three months among HIV-negative heterosexual men in AURAH and heterosexual men living with HIV in ASTRA.

	N=470 HIV-negative heterosexual men in AURAH^n^							N=373 HIV-positive heterosexual men in ASTRA						
		Any recreational drug use		Use of cannabis		Use of cocaine			Any recreational drug use		Use of cannabis		Use of cocaine	
	N	%	Age-adjusted^b^	%	Age-adjusted^b^	%	Age-adjusted^b^	N	%	Age-adjusted^b^	%	Age-adjusted^b^	%	Age-adjusted^b^
		*p-value*^a^	PR[95% CI]	*p-value*^a^	PR[95% CI]	*p-value*^a^	PR[95% CI]		*p-value*^a^	PR[95% CI]	*p-value*^a^	PR[95% CI]	*p-value*^a^	PR[95% CI]
			Overall *p-value*^c^		Overall *p-value*^c^		Overall *p-value*^c^			Overall *p-value*^c^		Overall *p-value*^c^		Overall *p-value*^c^
Age:														
<25	89	36.0%	3.06 [1.37, 6.82]	33.7%	2.87 [1.28, 6.42]	3.4%	0.32 [0.09, 1.14]	3	33.3%		33.3%		0.0%	
25-29	152	32.9%	2.80 [1.27, 6.14]	23.7%	2.01 [0.90, 4.50]	15.8%	1.49 [0.73, 3.06]	6	16.7%		16.7%		16.7%	
30-34	70	28.6%	2.43 [1.05, 5.62]	22.9%	1.94 [0.82, 4.62]	8.6%	0.70 [0.30, 1.66]^i^	20	15.0%	1.15 [0.49, 2.71]^k^	10.0%	1.20 [0.45, 3.21]^k^	10.0%	3.91 [1.03, 14.84]^k^
35-39	64	20.3%	1.73 [0.71, 4.23]	17.2%	1.46 [0.58, 3.69]	6.3%	1^j^	44	22.7%	1.52 [0.81, 2.84]	18.2%	1.59 [0.77, 3.28]	4.6%	1.72 [0.36, 8.26]
40-44	34	23.5%	2.00 [0.76, 5.26]	2.9%	1.46 [0.03, 1.99]	20.6%	*0.022*	63	17.5%	1.17 [0.63, 2.17]	11.1%	0.97 [0.44, 2.13]	3.2%	1.20 [0.25, 5.82]
45+	51	11.8%	1	11.8%	1	3.9%	*0.541*	227	15.0%	1	11.5%	1	2.6%	1
		*0.019*	*0.045*	*0.002*	*0.012*	*0.004*			*0.638*	*0.631*	*0.488*	*0.628*	*0.155*	*0.241*
		*<.001*	*<.001*	*<.001*	*<.001*	*0.884*			*0.313*	*0.318*	*0.300*	*0.361*	*0.157*	*0.078*
Ethnicity^o^:														
Black/m African	225	17.8%	1	15.1%	1	2.7%	1	188	5.9%	1	4.3%	1	0.5%	1
Black/m Caribbean	54	38.9%	2.04 [1.30, 3.22]	31.5%	1.91 [1.13, 3.20]	5.6%	/	27	33.3%	/	29.6%	\	3.7%	\
White	146	40.4%	2.15 [1.52, 3.03]	28.1%	1.74 [1.16, 2.60]	23.3%	8.60 [3.68, 20.09]	119	28.6%	5.40 [2.83, 10.31]	20.2%	5.23 [2.41, 11.34]	8.4%	20.04 [2.54, 158.02]
Other ethnicity	40	22.5%	1.22 [0.66, 2.28]	20.0%	1.26 [0.64, 2.48]	7.5%	2.39 [0.78, 7.29]^p^	26	19.2%	4.58 [2.20, 9.54]^p^	15.4%	5.40 [2.32, 12.58]^p^	3.9%	6.18 [0.61, 62.15]^p^
		*<.001*	*<.001*	*0.006*	*0.028*	*<.001*	*<.001*		*<.001*	*<.001*	*<.001*	*<.001*	*0.005*	*0.008*
London clinic site:														
Yes	350	29.7%	1	23.7%	1	11.1%	1	287	16.7%	1	12.5%	1	3.1%	m
No	120	21.7%	0.70 [0.48, 1.02]	15.0%	0.60 [0.37, 0.95]	6.7%	0.52 [0.24, 1.13]	86	14.0%	0.91 [0.51, 1.62]	10.5%	0.91 [0.46, 1.80]	4.7%	
		*0.089*	*0.062*	*0.045*	*0.03*	*0.158*	*0.101*		*0.539*	*0.402*	0.604	0.778	0.507	
University Education:														
Yes	274	22.6%	1	17.2%	1	7.7%	1	132	8.3%	1	5.3%	1	4.6%	1
No/missing	196	34.7%	1.54 [1.15, 2.06]	27.6%	1.60 [1.13, 2.26]	13.3%	1.71 [0.99, 2.96]	241	20.3%	2.51 [1.36, 4.65]	15.8%	3.06 [1.41, 6.63]	2.9%	0.67 [0.23, 1.93]
		*0.004*	*0.003*	*0.007*	*0.007*	*0.046*	*0.056*		*0.003*	*0.003*	*0.003*	*0.005*	*0.409*	*0.459*
Employed:														
Yes	340	25.9%	1	19.7%	1	11.2%	1	166	12.1%	1	9.6%	1	4.2%	1
No^d^/missing	130	32.3%	1.06 [0.76, 1.46]	26.2%	1.07 [0.73, 1.58]	6.9%	0.52 [0.24, 1.10]	207	19.3%	1.73 [1.05, 2.84]	14.0%	1.56 [0.88, 2.77]	2.9%	0.78 [0.27, 2.28]
		*0.164*	*0.737*	*0.128*	*0.725*	*0.169*	*0.088*		*0.057*	*0.032*	*0.198*	*0.125*	*0.49*	*0.656*
*(continued on next page)*

**Table 1:** (continued)

	N=470 HIV-negative heterosexual men in AURAH^n^							N=373 HIV-positive heterosexual men in ASTRA						
		Any recreational drug use		Use of cannabis		Use of cocaine			Any recreational drug use		Use of cannabis		Use of cocaine	
	N	%	Age-adjusted^b^	%	Age-adjusted^b^	%	Age-adjusted^b^	N	%	Age-adjusted^b^	%	Age-adjusted^b^	%	Age-adjusted^b^
		*p-value*^a^	PR[95% CI]	*p-value*^a^	PR[95% CI]	*p-value*^a^	PR[95% CI]		*p-value*^a^	PR[95% CI]	*p-value*^a^	PR[95% CI]	*p-value*^a^	PR[95% CI]
			Overall *p-value*^c^		Overall *p-value*^c^		Overall *p-value*^c^			Overall *p-value*^c^		Overall *p-value*^c^		Overall *p-value*^c^
Enough money^e^:														
Always	275	28.7%	1	21.5%	1	10.9%	1	106	10.4%	1	7.6%	1	1.89%	
Mostly	113	25.7%	0.88 [0.61, 1.28]	20.4%	0.93 [0.60, 1.45]	13.3%	1.14 [0.63, 2.07]	80	16.3%	1.51 [0.72, 3.19]	11.3%	1.44 [0.58, 3.56]	6.3%	1
At times/no	78	26.9%	0.98 [0.66, 1.47]	23.1%	1.13 [0.72, 1.77]	2.6%	0.25 [0.06, 1.00]	173	20.2%	1.90 [1.01, 3.59]	15.6%	2.02 [0.95, 4.28]	3.5%	0.88 [0.30, 2.55]^l^
		*0.818*	*0.81*	*0.903*	*0.782*	*0.042*	*0.115*		*0.098*	*0.133*	*0.131*	*0.166*	*0.259*	*0.809*
		*0.638*	*0.774*	*0.85*	*0.721*	*0.101*	*0.063*		*0.032*	*0.041*	*0.044*	*0.057*	*0.606*	
Housing:														
Home owner	71	21.1%	1	18.3%	1	5.6%	1	77	14.3%	1	10.4%	1	6.5%	m
Renting	286	26.2%	1.06 [0.65, 1.74]	18.9%	0.85 [0.49, 1.46]	11.2%	1.93 [0.72, 5.17]	213	15.5%	1.01 [0.53, 1.93]	12.7%	1.12 [0.52, 2.44]	1.9%	
Other^f^	106	35.9%	1.25 [0.73, 2.14]	30.2%	1.12 [0.62, 2.03]	9.4%	1.56 [0.50, 4.79]	69	21.7%	1.35 [0.66, 2.77]	13.0%	1.10 [0.45, 2.70]	5.8%	
		*0.068*	*0.568*	*0.042*	*0.340*	*0.368*	*0.392*		*0.405*	*0.528*	*0.850*	*0.957*	*0.368*	
		*0.024*	*0.332*	*0.034*	*0.445*	*0.524*	*0.625*		*0.236*	*0.398*	*0.618*	*0.837*	*1.000*	
Relationship^q^:														
Yes	302	19.5%	1	14.9%	1	7.0%	1	253	11.5%	1	8.7%	1	3.2%	1
No/missing	168	42.3%	1.91 [1.41, 2.59]	33.3%	1.92 [1.34, 2.74]	15.5%	2.30 [1.24, 4.26]	120	25.8%	2.33 [1.48, 3.66]	19.2%	2.28 [1.33, 3.90]	4.2%	1.42 [0.48, 4.21]
		*<.001*	*<.001*	*<.001*	*<.001*	*0.003*	*0.008*		*<.001*	*<.001*	*0.004*	*0.003*	*0.763*	*0.524*
High levels of social support^g^:														
Yes	330	27.6%	1	21.5%	1	10.6%	1	229	12.7%	1	10.5%	1	3.9%	1
No	120	31.7%	1.19 [0.87, 1.61]	24.2%	1.17 [0.81, 1.69]	10.0%	0.97 [0.52, 1.81]	126	23.8%	1.83 [1.15, 2.89]	15.9%	1.47 [0.85, 2.56]	3.2%	0.74 [0.23, 2.39]
		*0.396*	*0.278*	*0.550*	*0.412*	*0.853*	*0.936*		*0.007*	*0.010*	*0.140*	*0.172*	*0.717*	*0.618*
Current smoker:														
No/missing	367	19.1%	1	14.4%	1	6.3%	1	258	4.7%	1	2.3%	1	1.2%	1
Yes	103	58.3%	2.99 [2.29, 3.91]	46.6%	3.14 [2.26, 4.36]	23.3%	3.59 [2.11, 6.12]	115	41.7%	8.81 [4.87, 15.93]	33.9%	14.35 [6.25, 32.95]	8.7%	6.87 [1.93, 24.44]
		*<.001*	*<.001*	*<.001*	*<.001*	*<.001*	*<.001*		*<.001*	*<.001*	*<.001*	*<.001*	*<.001*	*0.003*
Higher-risk drinking^h^:														
No/missing	408	24.3%	1	19.6%	1	6.1%	1	329	14.6%	1	11.9%	1	2.7%	m
Yes	62	50.0%	1.89 [1.39, 2.57]	33.9%	1.56 [1.04, 2.34]	35.5%	5.92 [3.54, 9.92]	44	27.3%	1.88 [1.09, 3.24]	13.6%	1.15 [0.52, 2.56]	9.1%	
		*<.001*	*<.001*	*0.011*	*0.033*	*<.001*	*<.001*		*0.032*	*0.024*	*0.733*	*0.724*	*0.054*	

a p-value by pearson χ^2^ test or Fisher's exact test, followed by p-value for test for trend for ordered categorical variables (age, financial security, and housing).

b Adjusted for age as a continuous variable.

c Overall p-value for heterogeneity by Wald test in modified Poisson regression, followed by p-value for test for trend for ordered categorical variables (age, financial security, and housing).

d Including full time student, unemployed, permanently/temporarily sick, caretaker, retired.

e Enough money to cover basic needs (e.g. food, heating).

f Including unstable housing: temporary accommodation (hostel, shelter, bed and breakfast, squat), staying with partner/friend(s)/family, and homeless.

g Having a supportive network of individuals in one's life was assessed using a modified version of the Duke-UNC Functional Social Support Questionnaire (Broadhead, Gehlbach, de Gruy, & Kaplan, 1988) (with options: much less than I would like (1); less than I would like (2); some, would like more (3); almost as much as I would like (4); as much as I would like (5)). The total score across the five questions for each participant was generated. A total score of 21-25 was considered to indicate high levels of a supportive network and scores of 5-20 were considered to indicate lower levels of a supportive network.

h Higher-risk drinking is based on the first two questions of the WHO AUDIT-C questionnaire. Higher risk drinking is indicated by a score of ≥6 and lower risk drinking/no alcohol consumption by a score of <6. (Babor, Higgins-Biddle, Saunders, & Monteiro, 2001)

i Ages 30-39 years; combined age groups due to small cell counts.

j Age 40 plus years; combined age groups due to small cell counts.

k Ages <25 to 34 years; combined age groups due to small cell counts.

l Always/mostly enough money; combined categories due to small cell counts.

m Not calculated due to small cell counts.

n Individuals who had not been diagnosed with HIV at the time of the AURAH questionnaire completion. Four heterosexual men tested HIV-positive on the day of the AURAH questionnaire.

o Individuals reporting Black or mixed African ethnicity were categorised as Black/m African; those reporting Black or mixed Caribbean ethnicity were categorised as Black/m Caribbean. All white ethnic groups were categorised as white ethnicity and individuals from all other ethnic groups were categorised as other ethnicity.

p Black/m Caribbean and other ethnic groups; combined ethnic groups due to small cell counts.

q Participants were asked ‘Are you currently in an ongoing relationship with a partner (wife/husband or civil partner or girlfriend/boyfriend)?

**Table 2:** Unadjusted and adjusted associations of demographic, socio-economic and lifestyle factors with three measures of recreational drug use in the past three months among HIV-negative women in AURAH and women living with HIV in ASTRA.

	N=676 HIV-negative women in AURAH^n^							N=637 HIV-positive women in ASTRA						
		Any recreational drug use		Use of cannabis		Use of cocaine			Any recreational drug use		Use of cannabis		Use of cocaine	
	N	%	Age-adjusted^b^	%	Age-adjusted^b^	%	Age-adjusted^b^	N	%	Age-adjusted^b^	%	Age-adjusted^b^	%	Age-adjusted^b^
		*p-value*^a^	PR[95% CI]	*p-value*^a^	PR[95% CI]	*p-value*^a^	PR[95% CI]		*p-value*^a^	PR[95% CI]	*p-value*^a^	PR[95% CI]	*p-value*^a^	PR[95% CI]
			Overall *p-value*^c^		Overall *p-value*^c^		Overall *p-value*^c^			Overall *p-value*^c^		Overall *p-value*^c^		Overall *p-value*^c^
Age:														
<25	287	22.3%	3.20 [1.05, 9.73]	19.2%	2.64 [1.10, 6.36]	7.7%	5.29 [0.72, 38.62]	10	40.0%		20.0%		10.0%	
25-29	140	23.6%	3.38 [1.09, 10.5]	15.0%	2.07 [0.81, 5.26]	9.3%	6.41 [0.85, 48.06]	44	11.4%		11.4%		0.0%	
30-34	103	6.8%	0.97 [0.26, 3.60]	4.9%	1.08 [0.40, 2.92]^i^	2.9%	2.49 [0.31, 20.36]^i^	79	1.3%	1.11 [0.52, 2.39]^k^	0.0%	1.39 [0.53, 3.64]^k^	1.3%	1.19 [0.20, 7.03]^k^
35-39	63	15.9%	2.28 [0.66, 7.80]	12.7%	1^j^	4.8%	1^j^	98	6.1%	0.91 [0.37, 2.25]	4.1%	1.07 [0.34, 3.41]	3.1%	2.42 [0.50, 11.79]
40-44	26	7.7%	1.10 [0.20, 6.18]	7.7%	*0.005*	0.0%	*0.087*	134	6.7%	0.99 [0.45, 2.19]	4.5%	1.18 [0.43, 3.24]	1.5%	1.18 [0.20, 6.98]
45+	43	7.0%	1	7.0%	*<.001*	2.3%	*0.012*	237	6.8%	1	3.8%	1	1.3%	1
		*0.001*	*0.004*	*0.007*		*0.199*			*0.004*	*0.981*	*0.012*	*0.927*	*0.286*	*0.710*
		*<.001*	*0.001*	*0.001*		*0.025*			*0.242*	*0.855*	*0.157*	*0.553*	*0.514*	*0.582*
Ethnicity^o^:														
Black/m African	325	11.4%	1	10.2%	1	2.2%	1	420	1.0%	1	0.2%	1	0.5%	
Black/m Caribbean	114	21.9%	2.10 [1.33, 3.32]	21.1%	2.27 [1.40, 3.66]	4.4%	\	35	11.4%	\	11.4%	\	0.0%	m
White	163	30.1%	2.80 [1.92, 4.09]	19.0%	2.00 [1.28, 3.12]	16.6%	8.01 [3.58, 17.92]	124	24.2%	33.03 [10.23, 106.62]	16.1%	65.90 [8.95, 485.08]	5.7%	
Other ethnicity	65	13.9%	1.50 [0.77, 2.93]	10.8%	1.32 [0.61, 2.82]	4.6%	2.32 [0.86, 6.27]^p^	34	14.7%	15.86 [4.31, 58.29]^p^	8.8%	35.14 [4.28, 288.66]^p^	5.9%	
		*<.001*	*<.001*	*0.006*	*0.003*	*<.001*	*<.001*		*<.001*	*<.001*	*<.001*	*<.001*	*<.001*	
London clinic site:														
Yes	411	20.7%	1	16.6%	1	8.0%	1	505	6.3%	1	4.0%	1	1.8%	m
No	265	13.2%	0.61 [0.42, 0.88]	10.2%	0.59 [0.39, 0.90]	3.4%	0.40 [0.19, 0.82]	132	9.1%	1.57 [0.80, 3.07]	6.1%	1.61 [0.69, 3.78]	1.5%	
		*0.013*	*0.008*	*0.020*	*0.014*	*0.015*	*0.013*		0.267	0.191	0.295	*0.274*	*0.593*	
University Education:														
Yes	380	14.5%	1	11.1%	1	6.1%	1	198	3.5%	1	2.0%	1	1.0%	m
No/missing	295	22.0%	1.51 [1.09, 2.09]	18.0%	1.60 [1.10, 2.33]	6.4%	1.07 [0.59, 1.93]	439	8.4%	2.72 [1.16, 6.35]	5.5%	3.58 [1.09, 11.78]	2.1%	
		*0.011*	*0.013*	*0.010*	*0.013*	*0.836*	*0.826*		*0.024*	*0.021*	*0.050*	*0.036*	*0.517*	
Employed:														
Yes	361	18.6%	1	13.0%	1	8.3%	1	288	4.9%	1	3.47%	1	1.0%	1
No^d^/missing	315	16.8%	0.74 [0.52, 1.08]	15.2%	0.97 [0.64, 1.49]	3.8%	0.36 [0.17, 0.74]	349	8.6%	1.82 [0.96, 3.45]	5.2%	1.60 [0.73, 3.53]	2.3%	1.97 [0.51, 7.58]
		*0.556*	*0.116*	*0.408*	*0.899*	*0.016*	*0.006*		*0.064*	*0.065*	*0.302*	*0.245*	*0.228*	*0.321*
*(continued on next page)*

**Table 2:** (continued)

	N=676 HIV-negative women in AURAH^n^							N=637 HIV-positive women in ASTRA						
		Any recreational drug use		Use of cannabis		Use of cocaine			Any recreational drug use		Use of cannabis		Use of cocaine	
	N	%	Age-adjusted^b^	%	Age-adjusted^b^	%	Age-adjusted^b^	N	%	Age-adjusted^b^	%	Age-adjusted^b^	%	Age-adjusted^b^
		*p-value*^a^	PR[95% CI]	*p-value*^a^	PR[95% CI]	*p-value*^a^	PR[95% CI]		*p-value*^a^	PR[95% CI]	*p-value*^a^	PR[95% CI]	*p-value*^a^	PR[95% CI]
			Overall *p-value*^c^		Overall *p-value*^c^		Overall *p-value*^c^			Overall *p-value*^c^		Overall *p-value*^c^		Overall *p-value*^c^
Enough money^e^:														
Always	318	15.7%	1	11.3%	1	6.9%	1	133	6.8%	1	3.8%	1	2.3%	
Mostly	200	22.0%	1.37 [0.95, 1.96]	17.5%	1.51 [0.99, 2.31]	7.5%	1.06 [0.56, 1.99]	147	8.8%	1.46 [0.62, 3.41]	6.1%	2.01 [0.63, 6.37]	2.0%	1
At times/no	148	16.9%	1.10 [0.71, 1.71]	15.5%	1.41 [0.86, 2.29]	3.4%	0.52 [0.20, 1.36]	334	6.3%	0.97 [0.44, 2.17]	4.2%	1.32 [0.44, 3.96]	1.5%	0.58 [0.16, 2.03]^l^
		*0.181*	*0.228*	*0.123*	*0.140*	*0.243*	*0.358*		*0.595*	*0.473*	*0.570*	*0.430*	*0.775*	*0.392*
		*0.495*	*0.414*	*0.125*	*0.091*	*0.209*	*0.233*		*0.685*	*0.726*	*0.968*	*0.843*	*0.547*	
Housing:														
Home owner	155	9.5%	1	6.0%	1	6.0%	1	77	7.8%	1	6.7%	1	1.1%	m
Renting	682	19.7%	1.42 [0.69, 2.89]	15.7%	1.81 [0.74, 4.45]	6.8%	0.70 [0.25, 1.97]	213	7.9%	1.03 [0.45, 2.36]	4.6%	0.66 [0.26, 1.69]	2.4%	
Other^f^	292	17.7%	1.12 [0.52, 2.42]	14.5%	1.49 [0.58, 3.85]	5.4%	0.45 [0.13, 1.53]	69	2.7%	0.36 [0.10, 1.32]	2.7%	0.38 [0.10, 1.43]	0.0%	
		*0.087*	*0.325*	*0.067*	*0.314*	*0.792*	*0.372*		*0.156*	*0.195*	*0.412*	*0.355*	*0.231*	
		*0.268*	*0.597*	*0.171*	*0.992*	*0.712*	*0.157*		*0.134*	*0.064*	*0.184*	*0.133*	*0.468*	
Relationship^q^														
Yes	466	14.8%	1	11.8%	1	4.7%	1	346	7.8%	1	4.9%	1	2.3%	1
No/missing	210	24.3%	1.57 [1.14, 2.16]	19.1%	1.54 [1.07, 2.23]	9.5%	1.89 [1.04, 3.43]	291	5.8%	0.80 [0.43, 1.46]	3.8%	0.80 [0.37, 1.73]	1.0%	0.53 [0.13, 2.15]
		*0.003*	*0.006*	*0.012*	*0.021*	*0.017*	*0.036*		*0.331*	*0.461*	*0.487*	*0.577*	*0.216*	*0.372*
High levels of social support^g^:														
Yes	486	16.3%	1	12.4%	1	6.6%	1	370	6.5%	1	3.8%	1	1.9%	1
No	176	23.3%	1.44 [1.03, 2.01]	19.9%	1.61 [1.10, 2.36]	5.7%	0.89 [0.45, 1.77]	243	7.8%	1.28 [0.70, 2.36]	5.4%	1.46 [0.67, 3.19]	1.7%	1.05 [0.29, 3.77]
		*0.038*	*0.032*	*0.014*	*0.013*	*0.674*	*0.740*		*0.527*	*0.420*	*0.355*	*0.336*	*0.823*	*0.942*
Current smoker:														
No/missing	534	10.1%	1	7.5%	1	2.8%	1	570	2.3%	1	1.1%	m	1.1%	m
Yes	142	46.5%	4.28 [3.15, 5.81]	38.7%	4.79 [3.34, 6.87]	19.0%	6.39 [3.50, 11.65]	67	46.3%	18.3 [9.97, 33.46]	32.8%		7.5%	
		*<.001*	*<.001*	*<.001*	*<.001*	*<.001*	*<.001*		*<.001*	*<.001*	*<.001*		*0.003*	
Higher-risk drinking^h^:														
No/missing	643	16.2%	1	13.2%	1	4.5%	m	624	6.6%	m	4.2%	m	1.8%	m
Yes	33	48.5%	2.87 [1.94, 4.25]	30.3%	2.19 [1.26, 3.82]	39.4%		13	23.1%		15.4%		0.0%	
		*<.001*	*<.001*	*0.006*	*0.006*	*<.001*			*0.054*		*0.051*		*1.000*	

a p-value by pearson χ^2^ test or Fisher's exact test, followed by p-value for test for trend for ordered categorical variables (age, financial security, and housing).

b Adjusted for age as a continuous variable.

c Overall p-value for heterogeneity by Wald test in modified Poisson regression, followed by p-value for test for trend for ordered categorical variables (age, financial security, and housing).

d Including full time student, unemployed, permanently/temporarily sick, caretaker, retired.

e Enough money to cover basic needs (e.g. food, heating).

f Including unstable housing: temporary accommodation (hostel, shelter, bed and breakfast, squat), staying with partner/friend(s)/family, and homeless.

g Having a supportive network of individuals in one's life was assessed using a modified version of the Duke-UNC Functional Social Support Questionnaire (Broadhead et al., 1988) (with options: much less than I would like (1); less than I would like (2); some, would like more (3); almost as much as I would like (4); as much as I would like (5)). The total score across the five questions for each participant was generated. A total score of 21-25 was considered to indicate high levels of a supportive network and scores of 5-20 were considered to indicate lower levels of a supportive network.

h Higher-risk drinking is based on the first two questions of the WHO AUDIT-C questionnaire. Higher risk drinking is indicated by a score of ≥6 and lower risk drinking/no alcohol consumption by a score of <6. (Babor et al., 2001).

i Ages 30-39 years; combined age groups due to small cell counts.

j Age 40 plus years; combined age groups due to small cell counts.

k Ages <25 to 34 years; combined age groups due to small cell counts.

l Always/mostly enough money; combined categories due to small cell counts.

m Not calculated due to small cell counts.

n Individuals who had not been diagnosed with HIV at the time of the AURAH questionnaire completion. One woman tested HIV-positive on the day of the AURAH questionnaire.

o Individuals reporting Black or mixed African ethnicity were categorised as Black/m African; those reporting Black or mixed Caribbean ethnicity were categorised as Black/m Caribbean. All white ethnic groups were categorised as white ethnicity and individuals from all other ethnic groups were categorised as other ethnicity.

p Black/m Caribbean and other ethnic groups; combined ethnic groups due to small cell counts.

q Participants were asked ‘Are you currently in an ongoing relationship with a partner (wife/husband or civil partner or girlfriend/boyfriend)?

In total, 3258 people living with HIV participated in the ASTRA study (response rate was 64% of eligible patients approached), of whom 373 were heterosexual men and 637 were women. For heterosexual men and women respectively, 52.2% and 68.5% were of Black or mixed African ethnicity, 7.5% and 5.7% of Black or mixed Caribbean ethnicity, 33.1% and 20.2% of white ethnicity, and 7.2% and 5.6% of any other ethnicity. The median age was 47 (IQR 41–53) and 42 (IQR 35–48) respectively. The proportion of heterosexual men born in the UK, reporting a university degree level of education, and attending a clinic in London was 28.4%, 35.4%, and 76.9% respectively. The corresponding percentages for women were 18.7%, 31.1%, and 79.3% (Tables 1 and 2).

### Prevalence of recreational drug use

**Fig. 1:**
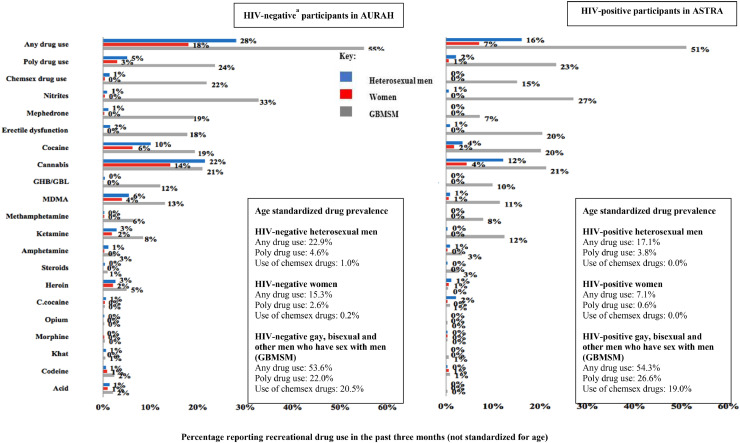
Prevalence of recreational drug use in the past three months (scale is to 60%). Figures show crude prevalence; age standardized prevalence for any drug use, poly drug use and use of drugs associated with chemsex are given in text boxes. Chemsex drug use = use of drugs (one or more of mephedrone, gamma-hydroxybutyrate/gamma-butyrolactone [GHB/GBL] and methamphetamine) associated with the practice of chemsex within gay communities^a^.

Prevalence of drug use is shown in Fig. 1. As the mean age was substantially younger for AURAH than ASTRA participants, the age-standardized prevalence of drug use is described here and also shown in Fig. 1 for the three main drug use measures. The prevalence of using at least one recreational drug in the past three months was 22.9% and 17.1% among HIV-negative and HIV-positive heterosexual men, and 15.3% and 7.1% among HIV-negative and HIV-positive women respectively. Five percent of HIV-negative heterosexual men and 3.8% of HIV-positive heterosexual men, and 2.6% of HIV-negative women and 0.6% of HIV-positive women reported poly drug use. For HIV-negative men, cannabis was the drug most commonly used in the past three months (17.9%, age standardized) followed by use of cocaine (8.5%) and MDMA (4.6%). For HIV-positive men, cannabis and cocaine were again the most prevalent (13.1% and 6.1% respectively), followed by crack cocaine (2.7%). Similarly, for HIV-negative women, cannabis (12.4%), cocaine (5.1%) and MDMA (3.0%) were the drugs most commonly used in the past three months. For HIV-positive women, cannabis (4.7%) and cocaine (1.6%) were the most commonly used drugs, with all other drugs having a prevalence of <1%. The prevalence of drug use associated with chemsex in gay communities in the past three months was very low among HIV-negative heterosexual men (1.0%) and women (0.2%) and was zero among HIV-positive heterosexual men and women.

The numbers of heterosexual men and women who reported injection drug use in the past three months were low: one HIV-negative heterosexual man, five HIV-positive heterosexual men, two HIV-negative women, and one HIV-positive woman.

### Comparison with GBMSM participants

To provide comparative information, Fig. 1 also shows the prevalence of recreational drug use among GBMSM participants. Prevalence of drug use overall, poly drug use, use of drugs associated with chemsex and use of most individual drugs was considerably higher among GBMSM compared to heterosexuals. Around a quarter of HIV-negative and HIV-positive GBMSM reported poly drug use, while 20.5% of HIV-negative and 19.0% of HIV-positive GBMSM reported use of drugs associated with chemsex (standardized for age).

### Socio-economic and lifestyle factors and recreational drug use among HIV-negative and HIV-positive heterosexual men

Among HIV-negative heterosexual men, younger age (<35 years), non-university degree level of education, being single, smoking, higher risk alcohol consumption and, to a lesser extent, London clinic site was associated with any drug use in unadjusted analysis and analysis adjusted for age. HIV-negative heterosexual men of Black/mixed Caribbean or white ethnicity were much more likely to report any drug use than men of Black or mixed African ethnicity, including after adjusting for age (Table 1). There was some indication of an association with more unstable housing but this was attenuated after age adjustment. Other socio-economic factors (employment, financial hardship, and supportive network) were not associated with drug use overall. The associations with cannabis use were similar. Again, a broadly similar pattern of associations was observed for cocaine use, although older rather than younger men were more likely to report using cocaine. In addition, financial security was associated with cocaine use in unadjusted, but not age-adjusted analysis (Table 1).

Among HIV-positive heterosexual men, non-university degree level of education, non-employment, being single, financial hardship, lower levels of a supportive network, smoking, and higher risk alcohol consumption was associated with any drug use in unadjusted and age-adjusted analysis. HIV-positive heterosexual men of white or any other ethnicity were much more likely to report any drug use than men of Black or mixed African ethnicity, including after adjusting for age (Table 1). The pattern of associations with cannabis was broadly similar to that found for any drug use, although employment, supportive network, and higher risk drinking was not associated with cannabis in unadjusted or age-adjusted analysis. There were fewer associations with cocaine use, in particular there was no relationship with education or financial hardship (Table 1).

### Socio-economic and lifestyle factors and recreational drug use among HIV-negative and HIV-positive women

Among HIV-negative women, younger age (<30 years), London clinic site, non-university degree level of education, being single, smoking, higher risk alcohol consumption, and lower levels of a supportive network was associated with any drug use in unadjusted and age-adjusted analysis. HIV-negative women of Black/mixed Caribbean or white ethnicity were much more likely to report any drug use than women of Black or mixed African ethnicity, including after adjusting for age (Table 2). Similar associations to that found for any drug use were observed with cannabis use and, with the exception of levels of education and a supportive network, with cocaine use. Being employed was also associated with cocaine use in unadjusted and age-adjusted analysis (Table 2).

Among women living with HIV, non-university degree level of education and smoking was associated with any drug use in unadjusted and age-adjusted analysis. HIV-positive women of white or any other ethnicity were much more likely to report any drug use than women of Black or mixed African ethnicity, including after adjusting for age (Table 2). Younger age (<25 years) was associated with any drug use in the χ^2^ analysis but the number of women in this age group was small and there was no association when combining age groups less than 35 in the unadjusted Poisson model. There was some evidence for an effect of non-employment and higher-risk alcohol consumption but the number of women who reported the latter was small. Similar associations to that found for any drug use were observed with cannabis use. For cocaine use, the pattern was broadly similar but there was no association with age, education and higher risk drinking.

### Recreational drug use and symptoms of depression and anxiety among HIV-negative and HIV-positive heterosexual men

**Fig. 2:**
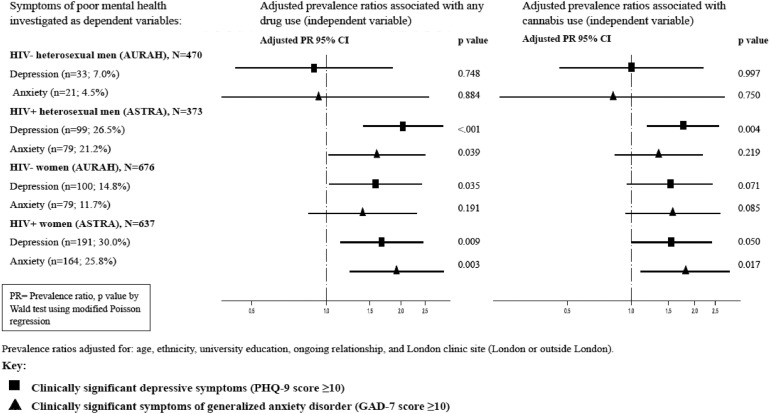
Adjusted associations of any drug use and cannabis use with depression and anxiety symptoms among HIV-negative and HIV-positive heterosexual men and women who participated in AURAH and ASTRA.

Among HIV-negative heterosexual men, the prevalence of symptoms of anxiety and depression was 7.0% and 4.5% respectively. No associations were observed for any drug use, cannabis use, or cocaine use with depressive symptoms or symptoms of anxiety in unadjusted or adjusted analysis (Fig. 2), although confidence intervals were wide. Among HIV-positive heterosexual men, symptom prevalence was much higher: 26.5% and 21.2% for depression and anxiety respectively. Any drug use and cannabis use was strongly associated with depressive symptoms in unadjusted and adjusted analysis. Any drug use but not cannabis use was associated with symptoms of anxiety in unadjusted and adjusted analysis (Fig. 2). Due to small numbers associations with cocaine use were not investigated.

### Recreational drug use and symptoms of depression and anxiety among HIV-negative and HIV-positive women

Among HIV-negative women, prevalence of symptoms was 14.8% for depression and 11.7% for anxiety. Any drug use was associated with depression in unadjusted and adjusted analysis. There was no significant association with anxiety (Fig. 2). Cannabis use was not associated with depression or anxiety among HIV-negative women. Cocaine use was not associated with depression or anxiety among HIV-negative women. Among HIV-positive women, there was a high prevalence of depression and anxiety symptoms (30.0% and 25.8% respectively). Any drug use and cannabis use was associated with both depressive and anxiety symptoms in unadjusted and adjusted analysis (Fig. 2). Cocaine use was not associated with depression or anxiety among HIV-positive women.

### Recreational drug use and sexual behavior and attitudes among HIV-negative and HIV-positive heterosexual men

Among HIV-negative heterosexual men, 43.4% reported CLS with a non-regular partner and just under a third reported CLS with two or more partners in the past three months. The prevalence of CLS with a non-regular partner was elevated among those who reported any drug use, cannabis use and cocaine use (Table 3). Only associations with cannabis and cocaine remained after adjustment for socio-demographic factors. The prevalence of CLS with two or more partners was elevated among those who reported any drug use and cannabis use (Table 3). The association with cannabis use was significant, and there was some evidence for the association with any drug use, in adjusted analysis. For the associations between any drug use and measures of CLS, the observed attenuation in adjusted analysis appeared to be driven primarily by adjustment for the relationship status measure; men not in an ongoing relationship were more likely to report recent drug use and CLS. Evidence of associations with low self-efficacy for sexual safety was found for any drug use and cocaine use in unadjusted analysis, but the association with any drug use was attenuated after adjustment. Two HIV-negative heterosexual men reported transactional sex for money or drugs in the past three months, neither of whom reported any drug use in the past three months.

**Table 3:** Unadjusted and adjusted associations of drug use measures with measures of CLS and sexual attitudes among HIV-negative heterosexual men and women in the AURAH study.

*N* = 470 heterosexual men^a^						
	CLS with non-regular partner (past 3 months) (*n* = 204; 43.4%)		CLS with two or more partners (past 3 months) (*n* = 144; 30.6%)		Lower self-efficacy for sexual safety (*n* = 660; 33.8%)	
	% *p-value*^a^	Adjusted^b^PR [95% CI]*p-value*^c^	% *p-value*^a^	Adjusted^b^PR [95% CI] *p-value*^c^	% *p-value*^a^	Adjusted^b^ PR [95% CI]*p-value*^c^
Any drug use No/missing Yes	39.1% 54.6% *0.002*	1 1.22 [0.99, 1.52] *0.065*	27.4% 39.4% *0.012*	1 1.33 [0.99, 1.78] *0.062*	31.5% 40.8% *0.057*	1 1.16 [0.88, 1.53] *0.301*
Use of cannabis No/missing Yes	39.6% 57.4% *0.001*	1 1.26 [1.02, 1.57] *0.036*	27.1% 43.6% *0.001*	1 1.48 [1.10, 2.00] *0.010*	33.1% 37.6% *0.391*	1 1.00 [0.73, 1.35] *0.977*
Use of cocaine No/missing Yes	41.1% 63.8% *0.003*	1 1.51 [1.17, 1.94] *0.001*	29.6% 40.4% *0.125*	1 1.37 [0.93, 2.03] *0.115*	32.2% 51.1% *0.009*	1 1.47 [1.04, 2.07] *0.028*

***N*** **=** **676 women**^a^						

	CLS with non-regular partner (past 3 months) (*n* = 192; 28.4%)		CLS with two or more partners (past 3 months) (*n* = 114; 16.9%)		Lower self-efficacy for sexual safety (*n* = 208; 30.8%)	
	% *p-value*^a^	Adjusted^b^ PR [95% CI] *p-value*^c^	% *p-value*^a^	Adjusted^b^ PR [95% CI] *p-value*^c^	% *p-value*^a^	Adjusted^b^ PR [95% CI] *p-value*^c^

Any drug use No/missing Yes	24.3% 47.5% *<0.001*	1 1.58 [1.24, 2.02] *<0.001*	12.8% 35.8% *<0.001*	1 2.24 [1.59, 3.17] *<0.001*	29.0% 39.2% *0.028*	1 1.44 [1.09, 1.91] *0.011*
Use of cannabis No/missing Yes	25.7% 45.3% *<0.001*	1 1.44 [1.10, 1.89] *0.008*	13.9% 34.7% *<0.001*	1 1.99 [1.38, 2.86] *<0.001*	29.6% 37.9% *0.105*	1 1.33 [0.98, 1.81] *0.070*
Use of cocaine No/missing Yes	26.3% 59.5% *<0.001*	1 1.75 [1.33, 2.30] *<0.001*	14.5% 52.4% *<0.001*	1 2.83 [1.97, 4.06] *<0.001*	29.8% 45.2% *0.036*	1 1.64 [1.12, 2.39] *0.011*

CLS= condomless sex.

a Individuals who had not been diagnosed with HIV at the time of the AURAH questionnaire completion. Four heterosexual men and one woman tested HIV-positive on the day of the AURAH questionnaire.

b Adjusted for: age (as a continuous variable), ethnicity (Black/mixed African or other ethnicity), university education, ongoing relationship status, and London clinic site.

c p-value by Wald test in modified Poisson regression.

Among HIV-positive heterosexual men, the prevalence of CLS measures was much lower. Thirteen individuals (3.5%) indicated CLS with a non-regular partner and 9 (2.4%) reported CLS with two or more partners in the past three months. Overall, 26.8% of HIV-positive heterosexual men reported low self-efficacy for sexual safety. There was an association between cocaine use (versus no cocaine use) and CLS with a non-regular partner (15.4% vs. 3.1%; *p* = 0.017). Measures of drug use were not associated with CLS with two or more partners or low self-efficacy for sexual safety in unadjusted analysis. Two men reported transactional sex for money or drugs in the past three months, neither of whom reported any drug use in the past three months.

### Recreational drug use and sexual behavior and attitudes among HIV-negative and HIV-positive women

Among HIV-negative women, 28.4% reported CLS with a non-regular partner and 16.9% reported two or more CLS partners in the past three months. Those who reported any drug use, cannabis use, and cocaine use were much more likely (approximately 1.5 to two-fold) to report CLS with a non-regular partner and two to three times more likely to report CLS with two or more partners, in unadjusted and adjusted analysis (Table 3). Women who reported any drug use and cocaine use were more likely to report low self-efficacy for sexual safety, in unadjusted and adjusted analysis. Seven HIV-negative women reported transactional sex for money or drugs in the past three months. Measures of any drug use, cannabis use, and cocaine use were all associated with an increased prevalence of transactional sex (for money or drugs) in univariable analysis: any drug use 4.2% vs. 0.4%; cannabis use 5.3% vs. 0.3%; cocaine use 4.8% vs. 0.8%; all p-values ≤0.01.

Among HIV-positive women, the prevalence of CLS measures was low. Thirteen women (2.0%) indicated CLS with a non-regular partner and 17 (2.7%) reported CLS with two or more partners in the past three months. Overall, 30.8% of women reported low self-efficacy for sexual safety. There was an association between any drug use (versus no drug use) and CLS with a non-regular partner (6.8% vs. 1.7%; *p* = 0.02), and between cocaine use and CLS with a non-regular partner (18.2% vs. 1.8%; *p*<0.001). Measures of drug use were not associated with CLS with two or more partners or low self-efficacy for sexual safety in unadjusted analysis. Two women reported transactional sex for money or drugs, both of whom reported drug use in the past three months (not cannabis nor cocaine).

## Discussion

This study assessed recreational drug use among HIV-negative heterosexual men and women recruited from sexual health clinics and HIV-positive heterosexual men and women recruited from HIV outpatient centres. Cannabis, cocaine, and among HIV-negative men and women, MDMA, were the most commonly used recreational drugs. Prevalence of use of drugs associated with chemsex in gay communities was very low in heterosexuals. A broadly similar pattern of associations with drug use was apparent across the four sample groups. Younger age, Black/mixed Caribbean and white ethnicity, attending a study clinic in London, non-university degree level of education, non-employment, being single, lower levels of a supportive network, smoking, and higher risk alcohol consumption tended to be associated with any recreational drug use and cannabis use. For cocaine use, the pattern of associations was broadly similar but notable differences were observed with age and socio-economic status. In some cases, cocaine use was more prevalent among older rather than younger people. Prevalence of cocaine use also tended to differ less between education groups, rather than being more prevalent in those with lower levels of educational attainment, and tended to be associated with employment and financial security. This may reflect the fact that cocaine has traditionally been popular as a stimulant among people in high-earning occupations (Johnston, O'Malley, Bachman & Schulenberg, 2008). In these heterosexual groups, there was evidence of associations of recreational drug use with measures of recent CLS, low self-efficacy for sexual safety and with symptoms of anxiety and depression. Associations with sexual behavior were particularly apparent among HIV-negative and HIV-positive women, and with poor mental health among HIV-positive men and women.

### Prevalence and correlates of recreational drug use among heterosexual men and women

Two UK population-based studies have collected data on recreational drug use: the Crime Survey for England and Wales (CSEW) and the National Survey of Sexual Attitudes and Lifestyles (Natsal). In the CSEW (2017/2018), the prevalence of any recreational drug use in the past year was 11.8% for men and 6.2% for women (ONSFlatley, 2018). In Natsal-3 (2010–2012), the prevalence of any recreational drug use in the past year among sexually active 16–44 year olds was 25.6% for men and 12.5% for women (Paquette et al., 2017). These current findings suggest that the prevalence of recreational drug use may be higher among heterosexuals attending sexual health clinics (28% in men and 18% in women; past three months) in England. When standardized by the age distribution in Natsal, the prevalence of any drug use among 16–44 year olds was 31.2% and 17.8% in HIV-negative men and women in AURAH respectively. The equivalent estimates were 23.1% and 17.2% in HIV-positive men and women in ASTRA. However, other socio-demographic factors may confound this comparison. There is a clear pattern across studies of heterosexual individuals in terms of the more common use of cannabis, followed by cocaine and/or MDMA (Bellis et al., 2008; ONSFlatley, 2018; Sumnall, Beynon, Conchie, Riley & Cole, 2007). This is in line with findings from the current study, for both HIV-negative and HIV-positive heterosexual individuals.

Studies of heterosexual men and women have found the following socio-economic and lifestyle correlates with recreational drug use: younger age (<20 years) (Hoare & Moon, 2010), white/mixed ethnicity (Paquette et al., 2017), lower levels of educational attainment (Morley, Lynskey, Moran, Borschmann & Winstock, 2015), not being employed (Morley et al., 2015), not being married/living alone (Hoare & Moon, 2010; Morley et al., 2015; Paquette et al., 2017), more frequent alcohol consumption (Hoare & Moon, 2010; Paquette et al., 2017), and smoking (Morley et al., 2015; Paquette et al., 2017). Drug use measures investigated included; any drug use (Hoare & Moon, 2010; Morley et al., 2015), drug use other than, or in addition to, cannabis (Paquette et al., 2017), use of cocaine/MDMA (Morley et al., 2015), and use of cannabis/MDMA (Morley et al., 2015), in the past year. Very similar associations were observed in the current study for HIV-negative and HIV-positive heterosexual men and HIV-negative and HIV-positive women, when examining cannabis, cocaine, and any drug use. In the current study, individuals of different Black ethnicities were analysed separately (Black/mixed African and Black/mixed Caribbean), as recent evidence in the UK suggests that sexual risk behaviors differ between Black ethnic groups (Coyle et al., 2018; Olonilua et al., 2008). Similarly, self-reported drug use prevalence was elevated in people of Black or mixed Caribbean ethnicity (and white ethnicity) compared to people of Black or mixed African ethnicity in the current study.

### Use of drugs associated with chemsex among heterosexual men and women

In the current UK study, the prevalence estimates of use of (one or more) drugs associated with chemsex in the past three months was very low for HIV-negative heterosexual men (1.3%) and women (0.3%). HIV-positive heterosexual men and women did not report use of drugs associated with chemsex. In contrast, about a fifth of GBMSM reported use of drugs associated with chemsex. Similar findings were observed in the Global Drug Survey (GDS), which recruited participants across 23 high-income countries via social media (2012). Among 11,577 heterosexual men, the prevalence of having sex while on methamphetamine, mephedrone, or GHB/GBL over the past year was 1.3%, 1.1%, and 0.7% respectively. The equivalent prevalence among 4970 heterosexual women was 0.9%, 1.3%, and 0.5%. Therefore, it does not appear that the phenomenon of chemsex is prevalent among heterosexuals. However, 29% of heterosexual men and 21% of heterosexual women in GDS reported ever having engaged in sexualized drug use (Lawn, Aldridge, Xia & Winstock, 2019). Other studies suggest that cannabis, MDMA, and cocaine may be used for sexual purposes among heterosexual individuals (Bellis et al., 2008; Sumnall et al., 2007). It is possible that the motives for sexualized use of drugs among heterosexuals may differ to those for chemsex among GBMSM. For instance, heterosexuals may take drugs to facilitate a sexual encounter or to relax more with a sexual partner (Bellis et al., 2008; Sumnall et al., 2007), whereas chemsex drugs may be taken by some GBMSM to further facilitate prolonged sessions (days in length) of intense sexual activity (Pakianathan, Lee, Kelly & Hegazi, 2016; Stuart, 2019).

### Association of recreational drug use with sexual behavior/attitudes and mental health symptoms among heterosexual men and women

Evidence from large high-income country studies and a meta-analysis suggest that among heterosexual men and women, any drug use, cannabis/MDMA, and cocaine/MDMA is strongly associated with CLS (Bellis et al., 2008; Brodbeck, Matter & Moggi, 2006; Morley et al., 2015; Paquette et al., 2017) and STI diagnosis (Paquette et al., 2017) in the past year. Associations are also apparent after adjustment for socio-demographic factors (Bellis et al., 2008; Morley et al., 2015; Paquette et al., 2017). Similar associations were observed in the current study for both HIV-negative and HIV-positive heterosexual men, and HIV-negative and HIV-positive women. Associations were particularly strong for women.

For HIV-negative men and women, cocaine use was associated with low self-efficacy for sexual safety (did not strongly agree ‘I feel confident that, if I want to, I can make sure a condom is used during sex with any partner, in any situation.’) in adjusted analysis. This is in line with a substantial body of theoretical work postulating that drug use, as well as alcohol use and depression, lowers one's self-efficacy for sexual safety, with implications for sexual risk taking (Bandura, 1997; Maciejewski, Prigerson & Mazure, 2000; Madden, 1991). For HIV-negative women, reporting any drug use was also associated with low self-efficacy for sexual safety in adjusted analysis. Women may be more susceptible to self-doubt and lack of assertiveness as a result of ingrained sexist norms (Abramson, 2014), and may be particularly vulnerable when under the influence of substances.

In a recent meta-analysis of seven studies (three U.S., two Australian, one Canadian, and one UK study), the pooled OR for the association between cannabis use during adolescence and depression in young adulthood was 1.37 (95% CI: 1.16, 1.62) (Gobbi et al., 2019). Based on findings from three studies, the pooled OR for the association with anxiety in young adulthood was 1.18 (95% CI: 0.84, 1.67). In the current study, similar associations were observed among HIV-positive heterosexual men and women. It is possible that these relationships may operate in the opposite direction such that depression/anxiety leads to recreational drug use, perhaps as a form of self-medication/escape from symptoms. Experiencing an HIV diagnosis and living with HIV may, for some people, encompass high levels of comorbid physical and psychological distress (Lampe et al., 2013). Cannabis has a long history of being used for therapeutic purposes in people living with HIV (Costiniuk & Jenabian, 2019), and there is some evidence from North American studies that medicinal cannabis use remains common in the modern ART era for managing comorbidities and alleviating symptoms of anxiety and depression (Belle-Isle & Hathaway, 2007; Harris et al., 2014). This may to some extent explain the strong associations observed between drug use and poor mental health symptoms among heterosexual men and women living with HIV in the current study, among whom the burden of depression and anxiety was greater.

### Strengths and limitations

Strengths of this study include the presentation of drug-specific prevalence estimates and their correlates among both HIV-negative heterosexuals and heterosexuals living with HIV in England, over a similar time period. The AURAH and ASTRA studies asked about recent recreational drug use, with a recall period of three months. Infrequent drug use might not be captured in these studies. However, the psychoactive effects of drug use may be immediate and short-lived. Investigating associations of recent recreational drug use with recent sexual behavior (also in the past three months) in this analysis might provide more accurate estimates of this relationship.

In terms of limitations, the cross-sectional methodology used in this study prohibits any inferences regarding causality. This study is important in the context of HIV-negative sexual health clinic attenders but these findings cannot necessarily be generalized to all heterosexual men and women in England, given the potentially differing behavioral profiles of these populations. The studies did not collect information specifically on sexualized drug use or on personality traits related to sensation-seeking/sexual compulsivity, childhood sexual abuse, and intimate partner violence, all of which may be important in the context of both recreational drug use and sexual risk behavior. Some analyses may have lacked power among male participants. Social desirability bias in the form of underreporting of drug use and CLS, particularly among HIV-positive participants, may have also affected these analyses, although all questionnaires were self-administered. Patterns of recreational drug use may have changed since the ASTRA and AURAH studies were conducted. Finally, the prevalence of pre-exposure prophylaxis (PrEP) use has likely increased substantially since the time of the AURAH study. Future studies will need to account for PrEP use in CLS measures and investigate whether recreational drug use is associated with non-adherence to PrEP. It is also crucial to note that HIV-positive people with an undetectable viral load cannot transmit HIV (Rodger et al., 2019), but CLS may still confer risk of STI transmission and acquisition.

## Conclusions

The prevalence of use of drugs associated with chemsex within gay communities was very low among HIV-negative and HIV-positive heterosexual men and women in the AURAH and ASTRA studies in England. However, findings indicate that health care providers need to be aware of the relatively high prevalence of cannabis, cocaine, and MDMA use among heterosexual men and women attending sexual health and HIV clinics, and the link with sexual activity and poor mental health symptoms. For HIV-negative women in particular, drug use may be a strong marker of sexual risk behavior. Although the prevalence of recreational drug use may be lower among heterosexual people living with HIV, special care should be afforded to those who do use drugs, in order to treat any possible underlying psychological/physical distress.

## Funding

This work was supported by the 10.13039/501100000272National Institute for Health Research (NIHR) under its Programme Grants for Applied Research funding scheme (RP-PG-0608–10142). The ASTRA and AURAH Study Groups acknowledge the support of the NIHR through the Comprehensive Clinical Research Network.

## Ethical approval

The AURAH study was approved by the NRES committee London-Hampstead (13/LO/0246). The ASTRA study was approved by the North West London REC 2 research ethics committee (10/H0720/70).

## Availability of data

We have a number of planned analyses for the AURAH and ASTRA study, but welcome proposals for additional analysis; please contact Dr Fiona Lampe (f.lampe@ucl.ac.uk). The Study Core Group will review proposals.

## Declarations of Interest

The authors declare that they have no known competing financial interests or personal relationships that could have appeared to influence the work reported in this paper.

## Footnotes

^a^ Individuals who had not been diagnosed with HIV at the time of the AURAH questionnaire completion. Four heterosexual men and one woman tested HIV-positive on the day of the AURAH questionnaire
